# The eating disorder examination questionnaire for adults from the Mexican general population: Reliability and validity

**DOI:** 10.1371/journal.pone.0266507

**Published:** 2022-04-07

**Authors:** José Alfredo Contreras-Valdez, Miguel-Ángel Freyre, Eleazar Mendoza-Flores

**Affiliations:** Facultad de Psicología, Universidad Nacional Autónoma de México, Mexico City, Mexico; University of Bologna, ITALY

## Abstract

The Eating Disorder Examination Questionnaire is a widely used self-report questionnaire for eating disorders. An Eating Disorder Examination Questionnaire model that is not lacking in relevant content has been supported in three different samples, but existing studies on this model present shortcomings regarding generalizations to the general population. Therefore, the general purpose of the current research was to test the reliability and interpretation validity of the Eating Disorder Examination Questionnaire 6.0 scores in adults of both sexes from the Mexican general population. After translating, adapting, and assessing the Eating Disorder Examination Questionnaire 6.0 in the target population through three pilot studies, we conducted two independent studies. In Study 1, 684 women and 433 men aged 18–83 participated, whereas in Study 2, 591 women and 382 men aged 18–86 did it. They answered the Eating Disorder Examination Questionnaire 6.0 and a measure of either body dissatisfaction (Study 1) or self-esteem (Study 2). According to confirmatory factor analyses, the 14-item Eating Disorder Examination Questionnaire model that we tested fit acceptably for the four samples (two female, two male) and was invariant across sex. All 14-item Eating Disorder Examination Questionnaire 6.0 scores were reliable according to Cronbach’s alpha and McDonald’s omega, except for only one factor score in men. Pearson’s correlations of the 14-item Eating Disorder Examination Questionnaire 6.0 scores with body dissatisfaction and self-esteem were positive and negative, respectively. This new Latin American Spanish translation of the Eating Disorder Examination Questionnaire 6.0 works broadly as expected and provides evidence to extend the generalization of previous studies to the general population. Thus, the present translation of the Eating Disorder Examination Questionnaire 6.0 may be a valuable tool in the field of eating disorders for researchers and practitioners studying or serving Latin American Spanish speakers of either sex from the general population.

## Introduction

In 1994, Fairburn and Beglin [1] published the Eating Disorder Examination Questionnaire (EDE-Q), which is a self-report questionnaire for measuring several symptoms of eating disorders. Its latest version, also known as EDE-Q 6.0 [2], has 22 items grouped into four dimensions, namely, Dietary Restraint, Eating Concern, Shape Concern, and Weight Concern [3]. This instrument was intended to aid in studying the validity of dimensional models of eating psychopathology and has become a widely used measure in this field of study [4–18].

In adults, research supporting the psychometric properties of the EDE-Q scores is broad. Regarding reliability, excellent values have been shown for both the test–retest method (Spearman’s correlations from .85 to .89 [19, 20]) and the intraclass correlation (.97 [21]). Nevertheless, Cronbach’s alpha has been the most common method used to examine the internal consistency of the EDE-Q scores, with coefficients for the overall scores usually ranging between .85 and .97 [4, 5, 7, 10, 13, 17, 18, 22–26]. Due to the limitations of this coefficient [27], it would be useful to study the reliability of the EDE-Q scores by applying more rigorous methods, such as McDonald’s omega, which is based on factor loadings from confirmatory factor analysis (CFA) [28], as some researchers have begun to do [12].

Regarding the validity of the interpretation of the EDE-Q scores, they have proven useful for discriminating adequately between patients with eating disorders and people without these diagnoses [4, 11, 17, 18, 23, 29–32]. In addition, the EDE-Q overall scores have shown positive correlations with other measures of eating disorders, for example, the SCOFF questionnaire, which measures the core features of anorexia nervosa and bulimia nervosa (*r*s = [.30, .44] [22]; *r*s = .70 [31]), and the Eating Attitudes Test, which is designed to measure dieting, bulimia, food preoccupation, and oral control (*r* = .75 [9]; *r* = .67 [30]; *r* = [.26, .74] [32]). However, the strongest correlations have been those with different versions of the Body Shape Questionnaire (BSQ), which measures body dissatisfaction (*r*s = [.65, .74] [22]; *r* = .88 [30]; *r* = [.59, .67] [33]; *r* = [.73, .89] [34]).

Additionally, the EDE-Q overall scores have also correlated with measures of other constructs, such as depression (*r* = [.15, .38] [6]; *r* = .24 [9]; *r*s = [.50, .61] [23]), anxiety (*r* = .27 [9]; *r*s = [.38, .47] [23]), and quality of life (*r*s = [−.53, −.27] [23]). Similarly, the EDE-Q overall scores have shown a positive correlation with low self-esteem (*r* = .34 [30]) and a negative correlation with high self-esteem (*r* = [−.32, −.10] [6]). These results are consistent with the transdiagnostic theory of eating disorders (the primary author of which is also the first author of the EDE-Q), which states that low self-esteem is a predictor of eating psychopathology [35].

Despite these results, the measurement model of the EDE-Q is still controversial. The original four-factor model [3] posed four dimensions based on the core features of eating behavior: diet, eating, shape, and weight. However, such model has received support from very few studies [21] in comparison with those not supporting it [4, 6, 11, 15, 22, 24, 30, 36–39].

In speakers of languages other than Spanish, through CFA it has been found that other models have shown a better fit. For example, Friborg et al. [38] found in women from the Norway general population a model of four specific factors (Shape Concern, Weight Concern, Eating Concern, and Dietary Restraint) that, in turn, were encompassed by an eating psychopathology general factor. Allen et al. [40] found in two samples of clinical and university women from Australia a moderate fit of an eight-item one-factor model. Kliem et al. [9] found in women and men from the Germany general population a fair fit for a model of eight items distributed among four factors corresponding to the original model [3]. Among those and other models, however, evidence has favored a model of seven items distributed among three factors (Dietary Restraint, Shape/Weight Overevaluation, and Body Dissatisfaction) that Grilo et al. [6] found in university students of both sexes in the United States, the fit of which has been shown to be adequate in other samples [4, 11, 12, 24, 41, 42].

Research on the EDE-Q in Spanish-speaking populations has presented results similar to those of research in speakers of other languages. For example, the original four-factor model [3] showed a moderate fit in Villarroel et al.’s [34] study in Spanish university women. However, Penelo et al. [43] did not find evidence for that model but a good fit for a two-factor model (Restraint and Eating–Shape–Weight Concern) in Mexican adolescents aged 11–18. The only one study on the EDE-Q 6.0 conducted in adults from Mexico found that the seven-item three-factor model by Grilo et al. [6] fit better than the original model [3] in both a sample of university women and a sample of women with eating disorders [26].

Although Grilo et al.’s model [6] has been the most replicated in various populations worldwide, it does not measure one factor originally proposed by Fairburn and colleagues [3], Eating Concern. After noting this limitation, Parker et al. [44, 45] found a four-factor model (Dietary Restraint, Eating Concern, Appearance Concern, and Shape and Weight Overevaluation) of 14 items in the United States. This model, which has been replicated in a recent study on university students of both sexes in the United States [15], would represent a better solution from a theoretical viewpoint because it would allow for a more complete measurement of the primary eating psychopathology symptoms.

Despite these supporting results and their promissory utility in measuring eating disorders, the existing studies present shortcomings regarding the generalization of their results. Notably, they have been focused on populations with specific features, such as people receiving bariatric surgery [44], candidates for bariatric surgery [45], and university students [15]. To date, no study has examined this model in a community or general population sample. Thus, the extant literature lacks generalizability to the population at large. Eating disorders have generally been conceptualized from a categorical perspective, which core assumption is that health is different from illness or disorder [46]. Several authors have observed problems emerging from this viewpoint both in the behavior field as a whole [47–50] and in the eating behavior domain [51]. The dimensional perspective states instead that a sharp difference between health and illness does not exist because behaviors, whether pathological or not, adaptive or not, comprise a continuum. Indeed, the “pathologicalness” or “adaptiveness” of any behavior is a matter of degree, not a feature; thus, any result of setting a criterion to determine who needs treatment (and who do not) is unavoidably arbitrary, and research based on those arbitrary decisions is likely biased [52, 53]. Including all population sectors would yield a full picture of the field, not just a partial profile comprising those that coincidentally fulfilled typified (arbitrary) criteria, which, in turn, would allow results to have more external validity. In the case of eating psychopathology, the general population has been found to present important symptom levels, and, though they are lower than in the clinical population, the way in which symptoms are expressed and related is similar [54]. Therefore, studying the general population (i.e., including all categorically healthy, categorically patient, and in-between subpopulations) with respect to Parker et al.’s model of the EDE-Q [44] is needed.

An additional reason for studying the general population was that the present research included translating and adapting the EDE-Q 6.0 to a different culture from the original. Thus, performing such a process in people from the general population would allow obtaining a seminal version that may be valid for most of the population, so that future studies in specific populations may be more accurate [16, 55].

Interestingly, those three studies that found evidence for Parker et al.’s model [44] have done so when combining women and men in their analyses [15, 44, 45]. Various studies have suggested that, in general, expression of eating psychopathology symptoms is still qualitatively different between sex groups. For example, shape, weight, and eating concerns are commonly expressed differently in men as compared to women [5, 18, 56]. Moreover, men are known to be less exposed to sociocultural pressure for a slim shape; the ideal masculine body is likely to be muscular and with low levels of fatty tissue [14, 56]. By contrast, women are subject to more pressure for achieving an unrealistic slim body [14], and their weight and shape are more related with their perception of personal worth [57]. In addition, it is true that research in this field has traditionally been focused on women, who frequently experience more symptom intensity [58]. Consequently, analyzing the sexes separately with Parker et al.’s model [44] and analyzing its invariance across sex are pending issues.

Thus, the general purpose of the current research was to test the reliability and interpretation validity of the EDE-Q 6.0 scores in adults of both sexes from the Mexican general population. To that aim, first, the instrument was translated and adapted; then, two independent studies were conducted. The rationale for conducting two separate studies was to add generalizability to conclusions from the present research by means of replication. Thus, in both studies similar aims were set, excepting that, to assess concurrent and convergent validity, the EDE-Q 6.0 scores were correlated with a measure of body dissatisfaction in Study 1 and self-esteem in Study 2.

## Translation and adaptation to adults from the Mexican general population

After receiving authorization via email to adapt the EDE-Q 6.0 to adults from the Mexican general population and test it (Mar 12, 2019 email from CG Fairburn to JA Contreras-Valdez; unreferenced, see “Acknowledgements”), two translators independently translated the text from English into Spanish. One of the translators was from the Escuela Nacional de Lenguas, Lingüística y Traducción Departamento de Traducción e Interpretación at the Universidad Nacional Autónoma de México. The other translator was not affiliated with the university.

The first author of the current research then back-translated the EDE-Q 6.0. To assess the suitability of the resulting version, three pilot studies were conducted. At the end of each one, linguistic adaptations were made to match the population features and needs. The third author and other 16 previously trained psychology undergraduates collected data in different places (e.g., schools, parks, plazas, hospitals, and homes) in Mexico City and the State of Mexico after the participants signed an informed consent form. Demographics are shown in Table 1. The final version was used in both studies reported below. The whole translation and adaptation process was conducted during May and June 2019.

**Table 1:** Demographic characteristics of study samples in women and men.

Characteristic	Pilot Study 1		Pilot Study 2		Pilot Study 3		Study 1		Study 2	
	Women	Men	Women	Men	Women	Men	Women	Men	Women	Men
*N*	49	24	41	22	36	27	684	433	591	382
Age (years old)										
Range	19–70	19–56	18–56	18–62	18–54	19–52	18–83	18–79	18–86	18–80
*M*	32	29	27	30	30	27	29	30	33	33
*SD*	14	13	11	12	11	9	13	12	14	14
*Mdn*	23	23	21	25	24	23	24	25	27	27
Highest educational level (% of group *n*) a										
None	—	—	—	—	—	—	0.15	0.00	0.85	0.52
Primary	—	—	—	—	—	—	1.61	1.15	4.57	2.62
Lower secondary	—	—	—	—	—	—	12.72	10.16	14.38	15.18
Upper secondary	—	—	—	—	—	—	58.48	56.12	51.44	50.79
Undergraduate studies	—	—	—	—	—	—	22.22	26.33	23.35	24.61
Postgraduate studies	—	—	—	—	—	—	4.24	5.58	4.57	5.50
Unknown	—	—	—	—	—	—	0.58	0.69	0.85	0.79

^a^ Data on the highest educational level were not collected for participants of pilot studies.

## Study 1

### Method

#### Aims and hypotheses

The Study 1 had four aims: (1) to test the fit of two measurement models separately in women and men, namely, the original four-factor model [3] and the four-factor model by Parker et al. [44]; (2) to examine invariance across sex in the best fitting model; (3) to determine the scores reliability by computing Cronbach’s alpha and McDonald’s omega; and (4) to assess the concurrent validity of the interpretation of the EDE-Q 6.0 scores through correlations with a measure of body dissatisfaction. Due to empirical support for Parker et al.’s model [44] in three different populations, the first hypothesis stated that this model would obtain a satisfactory fit. The second hypothesis suggested that invariance across sex would not be observed given that, if women and men differ qualitatively in their expression of eating psychopathology symptoms, then they would also do so quantitatively. The third hypothesis posed that the EDE-Q 6.0 scores would show adequate internal consistency through both methods. As a fourth hypothesis, a positive correlation between the EDE-Q 6.0 scores and the body dissatisfaction measure was expected.

#### Participants

A nonprobabilistic sample of 1,117 adults from the 16 districts of Mexico City and from 28 counties in the State of Mexico participated. Their demographics are shown in Table 1. The inclusion criteria were being 18 or older, having lived in Mexico City or the State of Mexico for at least 3 months before participating, and signing an informed consent form. The single exclusion criterion was presenting any condition that would hinder the ability to answer the instrument pack, such as being under the influence of alcohol or drugs or having a cognitive impairment. As an elimination criterion, incompletely answering the instrument pack was adopted. According to a dimensional theoretical perspective, having or not an eating disorder diagnosis was not a criterion for inclusion, exclusion, or elimination from the study. The initial sample comprised 1,301 participants, of whom 14.14% was eliminated, yielding the final total sample of 1,117. There were differences regarding sex between retained (684 women, 433 men) and eliminated participants (88 women, 78 men), χ^2^(1) = 4.08, *p* = .04, where the difference between retained and eliminated participants was greater in women (88.60% vs. 11.40%) than in men (84.74% vs. 15.26%). In addition, retained women (*n* = 684, *M* = 29, *SD* = 13) were younger than eliminated ones (*n* = 87, *M* = 33, *SD* = 13), *t*(769) = −2.63, *p* = .01; however, there were no age differences between retained (*n* = 433, *M* = 30, *SD* = 12) and eliminated men (*n* = 78, *M* = 30, *SD* = 12), *t*(509) = −0.18, *p* = .86.

#### Instruments

The EDE-Q 6.0 [2] was used once adapted to adults from the Mexican general population in the current investigation. It is a self-report instrument comprising 28 items, of which six have an open response format. Of the remaining 22 items, eight measure intensity over a continuum ranging from 0 to 6 and divided into four categories (*nada en absoluto* [not at all], *ligeramente* [slightly], *moderadamente* [moderately], and *totalmente* [completely]) without a definite correspondence between nonextreme categories and nonextreme points, whereas 14 measure frequency: 0 = *ningún día* [no days] or *ninguna vez* [none of the time], 1 = *1–5 días* [1–5 days] or *algunas veces* [sometimes], 2 = *6–12 días* [6–12 days] or *menos de la mitad de las veces* [less than half of the times], 3 = *13–15 días* [13–15 days] or *la mitad de las veces* [half of the times], 4 = *16–22 días* [16–22 days] or *más de la mitad de las veces* [more than half of the times], 5 = *23–27 días* [23–27 days] or *la mayoría de las veces* [most of the times], and 6 = *todos los días* [every day] or *siempre* [always]. In the current research, all 28 items were adapted and administered, although only the 22 seven-point items were considered in the analyses because they form the four original factors [3], and are thus the ones used in international research.

The BSQ [59] was also used. It is a 34-item self-report instrument with a 6-point response scale ranging from 1, *nunca* [never], to 6, *siempre* [always]. The 16-item version adapted to adolescents and adults from the Mexican population [60] was administered. Because the one-factor model did not obtain an adequate fit in the current research (women: χ^2^/*df* = 5.17, root-mean-square error of approximation [RMSEA] = .08, 90% confidence interval [CI] [.07, .08], standardized root-mean-square residual [SRMR] = .04, comparative fit index [CFI] = .92, nonnormed fit index [NNFI] = .90; men: χ^2^/*df* = 3.11, RMSEA = .07, 90% CI [.06, .08], SRMR = .05, CFI = .84, NNFI = .82), the eight-item one-factor model examined in previous studies [61–63] was used in the analyses. The scores showed adequate reliability, and the model fit to women’s (α = .91, ω = .92; χ^2^/*df* = 4.72, RMSEA = .07, 90% CI [.06, .09], SRMR = .03, CFI = .96, NNFI = .95) and men’s (α = .88, ω = .89; χ^2^/*df* = 2.34, RMSEA = .06, 90% CI [.04, .07], SRMR = .03, CFI = .95, NNFI = .94) data in the current study.

#### Procedure

The third author and 12 additional psychology undergraduates (six of those who assisted with the pilot studies and six new ones) approached potential participants directly in different places (e.g., schools, parks, plazas, hospitals, and homes); both recruitment places and prospects were freely chosen by undergraduates, who assessed fulfillment of the inclusion, exclusion, and elimination criteria by self-report or observation. Undergraduates invited prospects verbally, provided them with an informed consent form, and answered any question prospects asked. Afterwards, those fulfilling inclusion criteria, not fulfilling the exclusion criterion, and willing to participate were asked to recall three foods they had eaten in the last 28 days, using a print calendar as a visual aid. Then, the instruments were administered, either individually or collectively (for a maximum of five participants at once), in a paper-and-pencil format, and following the recommendations on administering the EDE-Q 6.0 [3]. Data collection was carried out during June and July 2019.

The current research adhered to the principles and standards of the *Código ético del psicólogo* [64] and the *Ethical Principles of Psychologists and Code of Conduct* [65]. The research project of which this study is part, Project IA302420, was approved by the Comité Evaluador del Área de las Ciencias Sociales and such approval was ratified by the Comité Técnico of the Universidad Nacional Autónoma de México Programa de Apoyo a Proyectos de Investigación e Innovación Tecnológica. None of these institutional review boards was specifically aimed at ethics, but they issued their approval based on a peer review of the project, including fulfillment of ethical standards.

Because the study involved only a survey to adults, the risk for the participants was very low. Moreover, the occurrence of any side effect from answering the instruments was also very unlikely, but we took three cautions: (1) all data collectors were trained to handle atypical situations that may arise during the data collection; (2) at the end of their participation, thinking about their benefit, the participants were given a list of the primary psychological care centers at the Universidad Nacional Autónoma de México; and (3) the participants were also offered the contact details of the principal investigator (i.e., the first author) in case someone wanted to express any comment or receive guidance. It should be noted that no participant reported distress regarding the instruments or contacted the principal investigator after their participation.

Additionally, according to the Declaration of Helsinki [66], the participants signed an informed consent form (by written) guaranteeing their anonymity and the confidentiality of their responses and answered voluntarily (i.e., for no obligation and without receiving any reward). Furthermore, we analyzed the data statistically, not at the individual level; therefore, participants’ identity was never unveiled in any way.

#### Data analysis

The fit of the original [3] and Parker et al.’s [44] measurement models were tested separately in women and men through CFA using the polychoric correlations matrix and the maximum-likelihood estimation method with robust standard errors and a Satorra–Bentler scaled test statistic. This approach was selected due to the lack of normality in the statistical distribution of the 22-item EDE-Q 6.0 overall score according to the Kolmogorov–Smirnov test in both women, *Z*(684) = 0.10, *p* < .001, and men, *Z*(433) = 0.13, *p* < .001. The lavaan package for RStudio (Version 4.1.1) was used. The analyses focused on the following fit statistics: RMSEA, SRMR, CFI, and NNFI. For a fit to be excellent, the values must be RMSEA ≤ .05, SRMR ≤ .05, CFI ≥ .95, and NNFI ≥ .95; to be acceptable, they must be RMSEA = (.05, .08], SRMR = (.05, .08], CFI = [.90, .95), and NNFI = [.90, .95). The chi-square statistic and the χ^2^/*df* ratio were included as descriptive information but were not considered due to their sample-size sensitivity and, in the case of the ratio, the arbitrariness of its cutoff point [67, 68]. All the CFAs were conducted without correlating the residual variances of the observed variables. The remaining analyses were performed only for the best fitting model.

By multiple-group CFAs, the EDE-Q 6.0 model invariance across sex was examined. Four nested models were sequentially analyzed progressively adding constraints. The same input matrix, estimation method, software, fit statistics, and cutoffs as before were used. Configural, metric, scalar, and residual invariance were tested. Models were compared using the differences in RMSEA and CFI, considering changes lower than |.01| to substantiate no differences [69].

Cronbach’s alpha and McDonald’s omega were computed to determine the internal consistency of the EDE-Q 6.0 overall and factor scores. The concurrent validity was calculated as the Pearson’s correlation of the EDE-Q 6.0 overall and factor scores with the BSQ overall score. For these analyses, IBM SPSS Statistics (Version 26) and the OMEGA macro, which allows McDonald’s omega to be obtained using IBM SPSS Statistics [70], were employed.

### Results

The original model [3] did not fit to data of either sex. By contrast, Parker et al.’s model [44] yielded an adequate fit for both women and men (see Table 2). The factor loadings and residual variances of this latter model may be found in Table 3, whereas Table 4 shows the factor covariances. Parker et al.’s model [44] showed full invariance across sex (see Table 5). The reliability of the 14-item EDE-Q 6.0 overall scores, which were adequate for both sexes, is provided in Table 6. For the factor scores, the reliability ranged from excellent to poor values according to both Cronbach’s alpha and McDonald’s omega. Regarding the concurrent validity, Table 7 presents the Pearson’s correlation coefficients between the 14-item EDE-Q 6.0 and BSQ scores. The coefficients were positive in both women and men.

**Table 2:** Results of the confirmatory factor analysis of two measurement models of the eating disorder examination questionnaire in women and men.

Model	χ^2^	*df*	χ^2^/*df*	RMSEA [90% CI]	SRMR	CFI	NNFI
Original model [3]							
Study 1							
Women (*n* = 684)	1,507.23***	202	7.46	.10*** [.09, .10]	.09	.76	.72
Men (*n* = 433)	758.13***	202	3.75	.08*** [.08, .08]	.09	.72	.68
Study 2							
Women (*n* = 591)	1,434.45***	202	7.11	.10*** [.10, .10]	.11	.74	.70
Men (*n* = 382)	728.77***	202	3.61	.08*** [.08, .09]	.10	.72	.68
Model by Parker et al. [44]							
Study 1							
Women (*n* = 684)	230.16***	71	3.24	.06 [.05, .06]	.04	.96	.94
Men (*n* = 433)	178.33***	71	2.51	.06 [.05, .07]	.05	.92	.89
Study 2							
Women (*n* = 591)	262.74***	71	3.70	.07*** [.06, .08]	.05	.94	.92
Men (*n* = 382)	156.01***	71	2.20	.06 [.05, .07]	.06	.92	.90

*Note*. RMSEA = root-mean-square error of approximation; CI = confidence interval; SRMR = standardized root-mean-square residual; CFI = comparative fit index; NNFI = nonnormed fit index.

***p < .001.

**Table 3:** Item statistics for Parker et al.’s model of the eating disorder examination questionnaire [44] in women and men.

Item	Study 1				Study 2			
	Women (*n* = 684)		Men (*n* = 433)		Women (*n* = 591)		Men (*n* = 382)	
	λ	ε	λ	ε	λ	ε	λ	ε
Dietary Restraint								
1. ¿En cuántos de los últimos 28 días has intentado a propósito limitar la cantidad de alimentos que comes, para modificar tu figura corporal o tu peso (ya sea que hayas tenido éxito o no)? [On how many of the last 28 days, have you deliberately tried to limit the amount of food you eat to modify your shape or weight (whether or not you have succeeded)?]	0.80	0.35	0.75	0.44	0.70	0.51	0.72	0.49
3. ¿En cuántos de los últimos 28 días has intentado quitar de tu dieta cualquier alimento que te guste para modificar tu figura corporal o tu peso (ya sea que hayas tenido éxito o no)? [On how many of the last 28 days, have you tried to exclude from your diet any foods that you like in order to modify your shape or weight (whether or not you have succeeded)?]	0.81	0.34	0.76	0.42	0.84	0.30	0.78	0.39
4. ¿En cuántos de los últimos 28 días has intentado seguir reglas definidas (fijas) con respecto a tu alimentación (por ejemplo, un límite de calorías) para modificar tu figura corporal o tu peso (ya sea que hayas tenido éxito o no)? [On how many of the last 28 days, have you tried to follow definite (fixed) rules regarding your eating (for example, a calorie limit) in order to modify your shape or weight (whether or not you have succeeded)?]	0.69	0.53	0.71	0.49	0.65	0.58	0.72	0.48
Eating Concern								
7. ¿En cuántos de los últimos 28 días pensar en los alimentos, las comidas o las calorías ha hecho que te sea muy difícil concentrarte en las cosas que estás interesado (por ejemplo, trabajar, tener una conversación o leer)? [On how many of the last 28 days, has thinking about food, eating or calories made it very difficult to concentrate on things you are interested in (for example, working, following a conversation, or reading)?]	0.47	0.78	0.40	0.84	0.39	0.84	0.41	0.84
9. ¿En cuántos de los últimos 28 días has tenido un temor fijo o persistente de perder el control sobre tu alimentación? [On how many of the last 28 days, have you had a definite or persistent fear of losing control over your eating?]	0.64	0.59	0.51	0.74	0.52	0.74	0.41	0.84
19. En los últimos 28 días, ¿en cuántos **días** has comido en secreto (a escondidas)? No cuentes episodios de atracón, ni las veces que has comido en secreto debido a las reglas de un lugar (por ejemplo, trabajo, escuela, etc.). [Over the last 28 days, on how many **days** have you eaten in secret (furtively)? Do not count episodes of binge eating or the times that you have eaten in secret because of the rules of a place (for example, work, school, etc.).]	0.45	0.80	0.43	0.82	0.64	0.60	0.64	0.60
20. En los últimos 28 días, ¿en qué **proporción** de las veces que has comido te has sentido culpable (has sentido que has hecho algo malo) debido a su efecto en tu figura corporal o peso? No cuentes los episodios de atracón. [Over the last 28 days, on what **proportion** of the times that you have eaten have you felt guilty (felt that you’ve done wrong) because of its effect on your shape or weight? Do not count episodes of binge eating.]	0.75	0.44	0.72	0.48	0.72	0.48	0.72	0.48
21. En los últimos 28 días, ¿**qué tan preocupada(o)** has estado de que otras personas te vean comer? No cuentes los episodios de atracón, ni las veces que te has preocupado de que te vean comer debido a las reglas de un lugar (por ejemplo, trabajo, escuela, etc.). [Over the last 28 days, **how concerned** have you been about other people seeing you eat? Do not count episodes of binge eating or the times that you have been concerned about other people seeing you eat because of the rules of a place (for example, work, school, etc.).]	0.72	0.48	0.62	0.62	0.71	0.50	0.61	0.63
Appearance Concern								
25. ¿En los últimos 28 días qué tan insatisfecha(o) has estado con tu **peso**? [Over the last 28 days, how dissatisfied have you been with your **weight**?]	0.81	0.34	0.71	0.50	0.84	0.30	0.77	0.41
26. ¿En los últimos 28 días qué tan insatisfecho(a) has estado con tu **figura corporal**? [Over the last 28 days, how dissatisfied have you been with your **shape**?]	0.89	0.21	0.80	0.37	0.89	0.20	0.82	0.33
27. ¿En los últimos 28 días qué tan incómoda(o) te has sentido al ver tu cuerpo (por ejemplo, al ver tu figura corporal en el espejo, en el reflejo de una ventana o escaparate, mientras te desvistes o al tomar un baño o una ducha)? [Over the last 28 days, how uncomfortable have you felt seeing your body (for example, seeing your shape in the mirror, in a window or shop window reflection, while undressing or taking a bath or shower)?]	0.93	0.13	0.86	0.26	0.85	0.28	0.86	0.27
28. ¿En los últimos 28 días qué tan incómodo(a) te has sentido acerca de que **otros** vean tu forma o figura corporal (por ejemplo, en los vestidores comunes, al nadar, o al traer ropa ajustada)? [Over the last 28 days, how uncomfortable have you felt about **others** seeing your figure or shape (for example, in communal changing rooms, when swimming, or wearing tight clothes)?]	0.89	0.21	0.87	0.25	0.84	0.29	0.80	0.37
Shape and Weight Overevaluation								
22. ¿En los últimos 28 días ha influido tu **peso** en lo que piensas acerca de ti (en cómo te juzgas) como persona? [Over the last 28 days, has your **weight** influenced what you think about yourself (how you judge yourself) as a person?]	0.92	0.16	0.89	0.21	0.84	0.29	0.91	0.16
23. ¿En los últimos 28 días ha influido tu **figura corporal** en lo que piensas acerca de ti (en cómo te juzgas) como persona? [Over the last 28 days, has your **shape** influenced what you think about yourself (how you judge yourself) as a person?]	0.95	0.09	0.88	0.23	0.90	0.19	0.89	0.22

*Note*. Translations into English were adapted from Fairburn and Beglin [2] to match the Spanish-language version after the slight modifications yielded by the instrument adaptation to adults from the Mexican general population. Items were adapted to the Mexican population and reproduced here with permission (Mar 12, 2019 and Apr 15, 2021 emails from CG Fairburn to JA Contreras-Valdez; unreferenced, see “Acknowledgements”). ε = residual variance.

**Table 4:** Factor covariances in Parker et al.’s model of the eating disorder examination questionnaire [44] in women and men.

Factor pair	Study 1		Study 2	
	Women (*n* = 684)	Men (*n* = 433)	Women (*n* = 591)	Men (*n* = 382)
1–2	.46***	.35***	.35***	.46***
1–3	.47***	.32***	.32***	.47***
1–4	.39***	.32***	.30***	.39***
2–3	.66***	.67***	.67***	.66***
2–4	.70***	.65***	.65***	.70***
3–4	.75***	.81***	.80***	.75***

*Note*. 1 = Dietary Restraint; 2 = Eating Concern; 3 = Appearance Concern; 4 = Shape and Weight Overevaluation.

***p < .001.

**Table 5:** Invariance of Parker et al.’s model of the eating disorder examination questionnaire [44] across sex.

Model	χ^2^	*df*	χ^2^/*df*	RMSEA [90% CI]	SRMR	CFI	NNFI	ΔRMSEA	ΔCFI
Study 1 (women *n* = 684, men *n* = 433)									
Configural	405.64***	142	2.86	.06*** [.05, .06]	.04	.94	.93		
Metric	402.80***	152	2.65	.05*** [.05, .06]	.04	.95	.93	−.01	.01
Scalar	426.93***	162	2.64	.05*** [.05, .06]	.04	.94	.93	.00	−.01
Residual	502.09***	176	2.85	.06*** [.05, .06]	.06	.93	.94	.01	−.01
Study 2 (women *n* = 591, men *n* = 382)									
Configural	402.19***	142	2.83	.06*** [.06, .07]	.05	.93	.91		
Metric	405.20***	152	2.67	.06*** [.05, .06]	.05	.93	.91	.00	.00
Scalar	437.58***	162	2.70	.06*** [.05, .07]	.05	.93	.92	.00	.00
Residual	444.31***	176	2.52	.06*** [.05, .06]	.06	.93	.93	.00	.00

*Note*. RMSEA = root-mean-square error of approximation; CI = confidence interval; SRMR = standardized root-mean-square residual; CFI = comparative fit index; NNFI = nonnormed fit index.

***p < .001.

**Table 6:** Internal consistency of factor scores of the 14-item eating disorder examination questionnaire 6.0 in women and men.

Factor	Study 1				Study 2			
	Women (*n* = 684)		Men (*n* = 433)		Women (*n* = 591)		Men (*n* = 382)	
	α	ω	α	ω	α	ω	α	ω
Overall score	.90	.90	.86	.86	.88	.88	.88	.88
Dietary Restraint	.81	.81	.78	.78	.77	.77	.78	.78
Eating Concern	.73	.75	.64	.65	.71	.71	.67	.66
Appearance Concern	.93	.93	.88	.88	.91	.91	.88	.88
Shape and Weight Overevaluation	.93	—	.87	—	.90	—	.89	—

*Note*. For the Shape and Weight Overevaluation factor, omega coefficients could not be computed due to the scarce number of items the factor includes. ω = McDonald’s omega coefficient.

**Table 7:** Pearson’s correlations among factors of the 14-item eating disorder examination questionnaire 6.0 (EDE-Q 6.0) and the body shape questionnaire in women (*n* = 684) and men (*n* = 433).

**Factor**	**1**	**2**	**3**	**4**	**5**	**6**	***M***	***SD***
1. EDE-Q 6.0 overall score		.66***	.76***	.88***	.79***	.84***	21	17
2. Dietary Restraint	.66***		.37***	.41***	.33***	.41***	5	5
3. Eating Concern	.70***	.31***		.54***	.57***	.64***	3	5
4. Appearance Concern	.85***	.31***	.50***		.69***	.82***	10	8
5. Shape and Weight Overevaluation	.76***	.30***	.47***	.65***		.72***	4	4
6. Body Dissatisfaction	.77***	.39***	.62***	.72***	.61***		19	9
*M*	15	4	2	6	2	15		
*SD*	13	5	3	6	3	7		

*Note*. Correlations in women are shown above diagonal, whereas correlations in men are presented below diagonal. Means and standard deviations for women are shown in the two last columns, and means and standard deviations for men are in the two last rows.

***p < .001.

## Study 2

### Method

#### Aims and hypotheses

As in Study 1, the second study also had four aims: (1) to test the fit of the original [3] and Parker et al.’s [44] measurement models separately in women and men; (2) to examine invariance across sex in the best fitting model; (3) to determine the scores reliability by computing Cronbach’s alpha and McDonald’s omega; and (4) to assess the convergent validity of the interpretation of the EDE-Q 6.0 scores through correlations with a self-esteem measure. Similar to Study 1, as the first hypothesis, Parker et al.’s model [44] was expected to show adequate fit in both sexes, whereas the original model [3] was expected not to fit. The second hypothesis suggested that no invariance would be found across sex. The third hypothesis posed that adequate reliability would be observed through both methods. The fourth hypothesis established that the EDE-Q 6.0 scores would correlate negatively with positive self-esteem and positively with negative self-esteem, accounting for the convergent validity of the interpretation of the EDE-Q 6.0 scores.

#### Participants

Through nonprobabilistic sampling, 973 adults from the 16 districts of Mexico City and from 27 counties in the State of Mexico participated. Their demographics are shown in Table 1. The inclusion, exclusion, and elimination criteria were the same as those in Study 1. From an initial sample of 1,101 participants, 11.63% was eliminated, yielding the final total sample of 973. There were no differences regarding sex between retained (591 women, 382 men) and eliminated participants (65 women, 42 men), χ^2^(1) = 0.00, *p* > .99, but retained women (*n* = 591, *M* = 33, *SD* = 14) were younger than eliminated ones (*n* = 65, *M* = 38, *SD* = 17), *t*(654) = −3.21, *p* = .001; in men, there were no age differences between retained (*n* = 382, *M* = 33, *SD* = 14) and eliminated participants (*n* = 42, *M* = 33, *SD* = 17), *t*(422) = −0.36, *p* = .85.

#### Instruments

The EDE-Q 6.0 [2], previously described, was used in this study, as well as the Rosenberg’s Self-Esteem Scale [71], adapted to the Mexican population [60, 72]. The Rosenberg’s scale is a self-report instrument comprising 10 items with a 4-point response scale: 1 = *totalmente de acuerdo* [completely agree], 2 = *de acuerdo* [agree], 3 = *en desacuerdo* [disagree], and 4 = *totalmente en desacuerdo* [completely disagree]. The model used for the analyses was the eight-item two-factor model by Jurado Cárdenas et al. [72], which has a Positive Self-Esteem factor composed of five items (women: α = .77, ω = .77; men: α = .80, ω = .80) and a Negative Self-Esteem factor integrated by three items (women: α = .77, ω = .78; men: α = .75, ω = .75). In the current study, the two-factor model showed adequate fit for women (χ^2^/*df* = 2.52, RMSEA = .05, 90% CI [.04, .07], SRMR = .04, CFI = .97, NNFI = .96) but poor fit for men (χ^2^/*df* = 2.99, RMSEA = .07, 90% CI [.06, .09], SRMR = .06, CFI = .91, NNFI = .86).

#### Procedure

The data collection was carried out by 11 psychology undergraduates (five of those who assisted with the pilot studies and six new ones). They administered the instruments the same way and in the same dates as in Study 1.

#### Data analysis

The Kolmogorov–Smirnov test showed that the 22-item EDE-Q 6.0 overall score did not have statistical normality in either women, *Z*(591) = 0.09, *p* < .001, or men, *Z*(382) = 0.13, *p* < .001, so the models fit and invariance across sex were examined with the same strategy used in Study 1. In addition, Cronbach’s alpha and McDonald’s omega were computed. Lastly, the convergent validity was assessed through the Pearson’s correlation of the EDE-Q 6.0 overall and factor scores with the scores for Rosenberg’s Self-Esteem Scale. For these analyses, IBM SPSS Statistics (Version 26) and the OMEGA macro [70] were used.

### Results

The CFAs revealed that the original model [3] did not fit to data of either sex. By contrast, Parker et al.’s model [44] presented adequate fit in both women and men (see Table 2). In Table 3, the factor loadings and residual variances are shown, whereas the factor covariances are displayed in Table 4. Notice in Table 5 that Parker et al.’s model [44] showed full invariance across sex. From Table 6, it may be appreciated that both Cronbach’s alpha and McDonald’s omega showed the 14-item EDE-Q 6.0 overall score to possess adequate reliability. The reliability of the factor scores ranged from adequate to doubtful values according to Cronbach’s alpha and McDonald’s omega. The results regarding the convergent validity are shown in Table 8. Overall, the Pearson’s correlations were as expected; that is, the 14-item EDE-Q 6.0 factor scores correlated positively with the Negative Self-Esteem factor and negatively with the Positive Self-Esteem factor. Only the Dietary Restraint factor showed no correlation with factors from the Self-Esteem Scale in women and showed almost no correlation in men.

**Table 8:** Pearson’s correlations among factors of the 14-item eating disorder examination questionnaire 6.0 (EDE-Q 6.0) and the Rosenberg’s self-esteem scale in women (*n* = 591) and men (*n* = 382).

**Factor**	**1**	**2**	**3**	**4**	**5**	**6**	**7**	***M***	***SD***
1. EDE-Q 6.0 overall score		.58***	.76***	.87***	.79***	−.42***	.26***	21	15
2. Dietary Restraint	.69***		.29***	.25***	.24***	−.07	−.01	5	5
3. Eating Concern	.72***	.34***		.56***	.52***	−.39***	.28***	3	4
4. Appearance Concern	.86***	.38***	.52***		.72***	−.41***	.26***	10	7
5. Shape and Weight Overevaluation	.81***	.42***	.49***	.69***		−.39***	.26***	4	4
6. Positive Self-Esteem	−.33***	−.10*	−.26***	−.37***	−.29***		−.48***	17	3
7. Negative Self-Esteem	.30***	.11*	.22***	.31***	.27***	−.60***		5	2
*M*	15	4	2	6	3	17	4		
*SD*	14	5	4	6	3	3	2		

*Note*. Correlations in women are shown above diagonal, whereas correlations in men are presented below diagonal. Means and standard deviations for women are shown in the two last columns, and means and standard deviations for men are in the two last rows.

*p < .05.

***p < .001.

## Discussion

The research reported here consisted of two studies. Both aimed first to separately test the fit of two measurement models of the EDE-Q 6.0 in women and men: the original four-factor model [3] and the four-factor model by Parker et al. [44]. The latter was expected to show an adequate fit for both sexes, whereas the former was not expected to fit. The results confirmed these expectations; that is, Parker et al.’s model [44] showed an acceptable fit, whereas the original model [3] did not. As reviewed in the introduction section, other studies have also supported Parker et al.’s model [15, 44, 45] or failed to find an acceptable fit for the original model [3] (e.g., [4, 6, 11, 15, 22, 24, 26, 30, 36–39, 43]). Parker et al.’s [44] showing an acceptable fit implies that this model might be correct enough to explain the relationships among the participants’ responses to the EDE-Q 6.0. Thus, from a practical point of view, researchers and practitioners may validly interpret the 14-item EDE-Q 6.0 scores as measures of four correlated dimensions underlying eating disorders. The first of these dimensions, Dietary Restraint, comprises behaviors aimed at modifying one’s shape or weight by controlling the amount, type, or content of food one eats. The second dimension, Eating Concern, addresses the association the person perceives between their eating and negative emotions (e.g., worry, fear, embarrassment, or guilt). The third dimension, Appearance Concern, covers the relationship of own shape or weight and unpleasant emotions (e.g., dissatisfaction or discomfort). The last of the four dimensions, Shape and Weight Overevaluation, asks about the influence that one’s shape or weight has on self-concept.

Additionally, from a theoretical point of view, the current results on model fit suggest that symptoms of eating disorders cluster not in accordance with very concrete features, such as food, shape, or weight, but in terms of psychological components: behavior, distress, unpleasantness, and cognition. Whilst the distinction between behavior and cognition may be clear, the distinction between distress and unpleasantness may not. Negative affect, or distress, is the affective dimension which high pole may be interpreted as anger, fear, guilt, stress, and so forth [73], any sort of alarm emotion. By contrast, unpleasantness is the negative pole of the valence dimension of affect, which may be construed as boredom, discomfort, dissatisfaction, sadness, and so forth [74], any simply aversive emotion not necessarily promoting action or preventing it. Therefore, although distress and unpleasantness may seem very similar or the same, they are not, and the divergence between them may explain better the difference found by Parker et al. [44] between the “concern” factors (i.e., Eating Concern and Appearance Concern) than extant explanations have done.

Another discrepancy that should be noted is the clustering of the shape- and weight-related symptoms. Although Fairburn [3] expected them to cluster according to shape on the one hand and weight on the other, subsequent research around the world have found them to cluster not consistently with that expectation [5, 6, 11, 15, 22, 24, 26, 30]. Parker et al. [44] explain the grouping as a concern, the one, and an overevaluation, the other. We further propose that the concern factor corresponds to an affective dimension whereas the overevaluation factor belongs to the cognitive domain.

If correct, the interpretation offered herein of Parker et al.’s [44] four-factor model of the EDE-Q strongly underlines the nature of eating disorders as psychological problems rather than primarily physical problems. Moreover, our interpretation also underscores the importance for researchers and practitioners to target their investigation, assessment, and treatment efforts at the affective, cognitive, and behavioral domains as the core of eating disorders, not at food, shape, or weight, which may be just the context or a superficial manifestation of the root problem, whatever it may be in each individual case.

The second aim of both studies was to examine the EDE-Q 6.0 model invariance across sex. Unexpectedly, in both studies the model was observed to be invariant, failing to support the posed hypothesis. The invariance analysis used in the present research is designed to detect whether the intended constructs have the same meaning for all groups under comparison [69]. Therefore, it seems that both women and men understood and answered in a similar way the 14 items included in Parker et al.’s model [44] of the EDE-Q. In addition, it should be noted that four levels of invariance were tested, namely, configural, metric, scalar, and residual. Parker et al.’s model [44] showed to be invariant throughout the four levels. From a practical point of view, these results imply that the 14-item EDE-Q 6.0 scores of women and men mean the same and thus may be safely compared to each other. Thus, researchers and practitioners may use the 14-item EDE-Q 6.0 scores in their studies and assessments regardless of the sex of their participants and patients.

In other respects, from a theoretical viewpoint, the invariance results obtained herein contradict four theoretical assumptions: (1) that women eating psychopathology may be radically different from that of men, which seems not the case because the model showed configural invariance; (2) that some symptoms may be more relevant for women than for men, but no, because the model showed metric invariance; (3) that women may have a higher baseline level or propensity for some or all eating psychopathology symptoms than men, but no, because the model showed scalar invariance; and (4) that there may be sex-specific factors that may contribute to some or all eating psychopathology symptoms, but no, because the model showed residual invariance. As Hyde [75] hypothesized, after all, women and men seem not to come from Venus and Mars, respectively; instead, both being from Earth are quite more similar to each other than different, even in regards to eating psychopathology as far as it concerns to the 14-item EDE-Q 6.0. Although contradicting the aforementioned theoretical assumptions, these invariance results are not alone: Previous studies have also found support for the EDE-Q being invariant across sex [6, 9, 33, 43], though considering other measurement models, not Parker et al.’s [44]. Therefore, another contribution of the present research is adding evidence on the EDE-Q invariance across sex but for the first time considering Parker et al.’s model [44].

The third aim of both studies was to determine the reliability of the EDE-Q 6.0 scores through the calculation of Cronbach’s alpha and McDonald’s omega. The EDE-Q 6.0 scores were posed to show adequate internal consistency through both methods. The results instead rectify that, as expected, three dimensions of Parker et al.’s model [44], namely, Dietary Restraint, Appearance Concern, and Shape and Weight Overevaluation, were internally consistent according to both Cronbach’s alpha and McDonald’s omega. The Eating Concern dimension, however, was not consistent enough. Thus, on the one hand, from a practical viewpoint, researchers and practitioners may rely on the 14-item EDE-Q 6.0 scores, except for the Eating Concern one, when assessing symptom dimensions of eating disorders. On the other hand, from a theoretical viewpoint, these results suggest that, except for Eating Concern, each of the dimensions of Parker et al.’s model [44] is homogenous enough to constitute a true dimension or construct. In other words, each dimension may be interpreted as a consistent factor underlying the symptoms of eating disorders.

The Eating Concern dimension not being reliable indicates that behaviors included in the Eating Concern dimension are not similar enough to each other to measure a single factor. These emotions may cluster into further dimensions or combine with other dimensions, whether considered or not in Parker et al.’s model [44]. Another possibility is that not emotions but rather an unknown factor influencing participant responses is causing the lack of consistency within the Eating Concern dimension. Future studies should examine this issue in more detail. Meanwhile, researchers and practitioners should interpret with caution this dimension.

As all or almost all former studies have tested, the reliability of the overall scores was also examined in both current studies, for comparative purposes, even if these scores were not posed in Parker et al.’s model [44] as dimensions. Specifically, scores based on the 14 items comprised in Parker et al.’s model [44] of the EDE-Q 6.0 were tested for internal consistency. The score succeeded in the Cronbach’s alpha and McDonald’s omega tests. Previous studies have also arrived at the same conclusion through several methods that various EDE-Q overall scores (i.e., varying in number of items) are reliable [4, 5, 7, 10, 12, 13, 17–26]. Thus, as with the three factor scores mentioned above, both overall scores may be used and interpreted confidently as dimensions. Whether such general dimensions may replace the four specific dimensions or otherwise what role those general dimensions may play (a second-order role, a parallel role, etc.), what they may mean in research and practical contexts (eating psychopathology or something else), whether eating psychopathology or whatever those dimensions measure may be conceptualized as a single continuum, and so forth, are pending questions. Until those and related issues are not solved empirically, the use and interpretation of the 14-item EDE-Q 6.0 overall score should be avoided or performed cautiously.

The fourth aim of both studies was to assess the validity of the interpretation of the EDE-Q 6.0 scores regarding their correlations with other measures. Specifically, in Study 1, the concurrent validity was analyzed as a function of the correlations of the 14-item EDE-Q 6.0 scores with a measure of body dissatisfaction, whereas in Study 2, the convergent validity was addressed as correlations of the 14-item EDE-Q 6.0 scores with a self-esteem measure. A positive correlation was expected with body dissatisfaction, and a negative correlation was expected with self-esteem. The results supported these assumptions, because the 14-item EDE-Q 6.0 scores positively correlated with body dissatisfaction and negative self-esteem but negatively correlated with positive self-esteem. These results are consistent with previous studies [6, 22, 30, 33, 34]. In addition, all the correlations with body dissatisfaction were strong or moderate, except those with Dietary Restraint, which were just moderate. Correlations with self-esteem were moderate with positive self-esteem and rather weak with negative self-esteem, except for the correlations of Dietary Restraint, which may be better understood as null. From a practical perspective, all these correlations corroborate two facts. On the one hand, symptoms of eating disorders may be present at the same time as body dissatisfaction, so when severe or frequent symptoms are detected, especially cognitive and affective symptoms, one is likely to also find frequent body dissatisfaction, and conversely, to find severe or frequent symptoms may be expected when frequent body dissatisfaction is detected. On the other hand, the severity and frequency of the cognitive and affective symptoms of eating disorders may vary somewhat with the self-esteem level, so as the symptom severity and frequency increase, the self-esteem level may decrease slightly, and the reverse, as the symptom severity and frequency decrease, self-esteem may increase slightly. In this example, mentioning symptoms first is not meant to state any causal relationship, because correlations do not bring evidence of causality, so to say that as self-esteem increase or decrease, symptoms may slightly decrease or increase, respectively, is equally true according to the results reported here.

Moreover, from a theoretical perspective, the correlations found in the current studies suggest that researchers and practitioners may validly interpret the 14-item EDE-Q 6.0 scores as measures of symptoms of eating disorders that may be present both together with body dissatisfaction and in accordance with self-esteem. The fact that correlations of the behavioral dimension, Dietary Restraint, were weaker with both body dissatisfaction and self-esteem than correlations of the affective and cognitive dimensions makes sense because body dissatisfaction and self-esteem are constructs that are heavily loaded with affective and cognitive responses rather than with instrumental responses, so it should not be surprising to find stronger correlations among emotions and cognitions than between them and instrumental behaviors. Indeed, according to Fairburn et al. [35], cognitions and emotions about eating (i.e., concerns about food, shape, and weight) precede the eating instrumental behaviors (i.e., dietary restraint), so these factors are not all at the same level. Future longitudinal studies should test a more theoretically congruent model stating these two different moments in the development of eating disorders. In addition, given that the 14-item EDE-Q 6.0 scores relationships with body dissatisfaction are stronger than its relationships with other measures of eating disorders raises the issue of whether the EDE-Q 6.0 truly measures symptoms of eating disorders or rather body dissatisfaction. Regarding the content of the EDE-Q 6.0 items, an affirmative answer to this question seems unlikely, but future studies should provide clarity. Regarding self-esteem, the fact that the correlations were not strong, especially with negative self-esteem, questions to what extent self-esteem truly predicts eating psychopathology, as posed in the transdiagnostic theory of eating disorders [35]. The more experimental future studies are, the greater they may aid in clarifying this issue.

As stated in former paragraphs, the results from the current research are basically consistent with previous studies, but they do so in two samples of a wider population array and separately for women and men, so the results come from four independent samples or, in what may be the same configuration, a testing sample and three replicas. Therefore, one strength of the studies reported here is the consistency of results across samples. A second strength is the heterogeneity within each sample, because the participants greatly varied regarding at least age, schooling, and residency; Mexico City and its courban area of the neighboring State of Mexico comprise a rather large and economically varied location.

The current studies also have some limitations. The sampling method was not random, so sample representativeness was not assured. The participants come from a single city, so generalization may be judged as limited if not considered in the context of the research line to which this paper contributes. Some inclusion or exclusion criteria (i.e., being 18 or older, having lived in the selected locations for at least 3 months before participating, and presenting any condition that would hinder the ability to answer the instrument pack) were partially or totally assessed by self-report, so their fulfillment cannot be warranted. As well, the measures were all retrospective self-reports, so biases from social desirability, reading skills, tiredness, memory functioning, and so on cannot be ruled out. The data were collected cross-sectionally, precluding any test of predictive validity or temporal stability. Besides, a more complete view of the EDE-Q 6.0 nomological network is missing, because having used only one related measure in each study has given initial, though limited information. Contrasts across samples or models were not performed, so the comparisons attempted here are only *de facie*. Future studies should overcome these limitations by granting a random sample, recruiting people from multiple locations, assessing inclusion and exclusion criteria through documents and other objective evidence, including several measures, preferably behavioral, neuroimaging, or biochemical measures, adopting a longitudinal design, or conducting analyses of direct group comparisons to mention the most evident improvements.

Despite these limitations, the current research provides evidence on a Spanish-language version of the EDE-Q 6.0 that was carefully translated, adapted, and assessed in the target population through three pilot studies, which was not performed for the Mexican population before. Another noteworthy contribution is that the studies reported here included a wider array of the population than previous studies on Parker et al.’s model [44]; that is, the participants came from the community at large instead of from populations with specific features (e.g., people receiving bariatric surgery, candidates for bariatric surgery, or university students [15, 44, 45]). Therefore, replicating the previous results supports their generalization to the general population. In addition, given that the results reported in this paper are consistent with studies conducted in populations other than Mexicans, the generalization of the results may not be limited to the Mexican general population but rather extend to the general population of several nations. Naturally, this last conjecture awaits empirical examination. Moreover, equivalence across sex seems likely. At least, however, the current results allow us to state that the 14-item EDE-Q 6.0 scores may be reliable and validly interpreted in both women and men. Thus, this paper furthers knowledge about Parker et al.’s model [44], given that previous studies have not accounted for sex. Lastly, the current research was conducted in Mexico, being one of the few to do so until now, and thus adding to global knowledge on measuring symptoms of eating disorders through the EDE-Q 6.0.

In summary, in this paper, we aimed to test the reliability and interpretation validity of the EDE-Q 6.0 scores in adults of both sexes from the Mexican general population. A new Latin American Spanish translation was introduced and tested for two basic aspects: its internal structure and its relationship to measures of similar constructs, namely, body dissatisfaction and self-esteem. Both primary studies provided evidence for the reliability of and interpretation validity of the EDE-Q 6.0 scores derived from Parker et al.’s model [44]. Thus, it seems acceptable to conclude that this translation of the EDE-Q 6.0 works broadly as expected and provides evidence to extend the generalization of previous studies to the general population. The present translation of the EDE-Q 6.0 may be a valuable tool in the field of eating disorders for researchers and practitioners studying or serving Latin American Spanish speakers, particularly Mexicans, of either sex from the general population.

## Supporting information

S1 Dataset Studies 1 and 2 data. (XLSX)
